# The effect of polydispersity, shape fluctuations and curvature on small unilamellar vesicle small-angle X-ray scattering curves

**DOI:** 10.1107/S1600576721001461

**Published:** 2021-03-25

**Authors:** Veronica Chappa, Yuliya Smirnova, Karlo Komorowski, Marcus Müller, Tim Salditt

**Affiliations:** a Faculty of Physics, University of Göttingen, Friedrich-Hund-Platz 1, Göttingen 37077, Germany

**Keywords:** small unilamellar vesicles, coarse-grained simulations, elastic simulations, small-angle X-ray scattering, SAXS curves

## Abstract

Small unilamellar vesicles (20–100 nm diameter) are model systems for strongly curved lipid membranes, in particular for cell organelles. Routinely, small-angle X-ray scattering (SAXS) is employed to study their size and electron-density profile (EDP). Current SAXS analysis of small unilamellar vesicles (SUVs) often employs a factorization into the structure factor (vesicle shape) and the form factor (lipid bilayer electron-density profile) and invokes additional idealizations: (i) an effective polydispersity distribution of vesicle radii, (ii) a spherical vesicle shape and (iii) an approximate account of membrane asymmetry, a feature particularly relevant for strongly curved membranes. These idealizations do not account for thermal shape fluctuations and also break down for strong salt- or protein-induced deformations, as well as vesicle adhesion and fusion, which complicate the analysis of the lipid bilayer structure. Presented here are simulations of SAXS curves of SUVs with experimentally relevant size, shape and EDPs of the curved bilayer, inferred from coarse-grained simulations and elasticity considerations, to quantify the effects of size polydispersity, thermal fluctuations of the SUV shape and membrane asymmetry. It is observed that the factorization approximation of the scattering intensity holds even for small vesicle radii (∼30 nm). However, the simulations show that, for very small vesicles, a curvature-induced asymmetry arises in the EDP, with sizeable effects on the SAXS curve. It is also demonstrated that thermal fluctuations in shape and the size polydispersity have distinguishable signatures in the SAXS intensity. Polydispersity gives rise to low-*q* features, whereas thermal fluctuations predominantly affect the scattering at larger *q*, related to membrane bending rigidity. Finally, it is shown that simulation of fluctuating vesicle ensembles can be used for analysis of experimental SAXS curves.

## Abstract 2

It is a challenge to distinguish the effect of shape fluctuations and size polydispersity on experimental small-angle X-ray scattering curves of small unilamellar vesicles. Here it is shown that both effects have distinguishable spectral patterns, and an efficient simulation tool is presented for simulating and analysing experimental data. The importance of curvature-induced electron-density profile asymmetry for estimating the vesicle size from SAXS scattering curves is also demonstrated.

## 1. Introduction   

The shape of fluctuating membranes has received abiding attention in the context of measuring bending rigidity (Gompper & Kroll, 1997 ▸) and explaining frequent, but surprising, membrane shapes such as the discoid shape of red blood cells (Canham, 1970 ▸; Helfrich, 1973 ▸; Seifert *et al.*, 1991 ▸; Discher *et al.*, 1994 ▸; Safran, 1994 ▸; Lim *et al.*, 2002 ▸; Li *et al.*, 2005 ▸). One of the most widely used experimental techniques to study the structure and shape of lipid bilayers and vesicles is small-angle X-ray scattering (SAXS) (Zubay, 1999 ▸; Pabst *et al.*, 2000 ▸; Kiselev *et al.*, 2002 ▸; Brzustowicz & Brunger, 2005 ▸; Pencer *et al.*, 2006 ▸; Kučerka *et al.*, 2008 ▸; Székely *et al.*, 2010 ▸; Heberle *et al.*, 2012 ▸). It allows information to be obtained starting from the molecular level, such as the location of a particular chemical entity (*e.g.* carbon double bonds), up to the overall vesicle shape. However, a quantitative analysis of experimental scattering data that includes the effects of (i) size polydispersity, (ii) vesicle shape fluctuations and (iii) the curvature-dependent electron-density profile (EDP) has remained challenging.

Theoretical methods, such as molecular dynamics (MD) simulations (Marrink *et al.*, 2009 ▸) and continuum elastic vesicle models (Gompper & Kroll, 1996 ▸), can help to interpret and understand experimental data by analysing contributions from different physical factors. For small unilamellar vesicles (SUVs) with radii in the range of 10–50 nm, such methods as coarse-grained MD simulations and the elastic Helfrich (1973 ▸, 1986 ▸) model can be used efficiently. SUVs are also valuable model systems for studying membrane adhesion and fusion (Komorowski *et al.*, 2018 ▸). It is therefore worthwhile to combine simulation techniques and SAXS in order to better understand small unilamellar vesicle shapes and their membrane EDPs. In this work, we use a hierarchical simulation framework that employs different models and simulation techniques on different scales (Müller *et al.*, 2003 ▸; Müller *et al.*, 2006 ▸): On the scale of the bilayer thickness, we obtain a curvature-dependent radial EDP of a lipid membrane from MD simulations using the coarse-grained MARTINI force field (Marrink *et al.*, 2007 ▸). We then use this profile to dress various vesicle shapes generated using the elastic Helfrich model (Seifert, 1997 ▸). The resulting three-dimensional electron-density map is used to calculate the scattering intensity via a three-dimensional (3D) fast Fourier transform (FFT) and subsequent powder averaging, taking into account many realizations for an ensemble averaging over thermal shape fluctuations and size polydispersity. We compare the simulation results of our numerical model with existing analytical SAXS models (Brzustowicz & Brunger, 2005 ▸), and demonstrate its capability to be used for least-squares fitting of experimental SAXS curves obtained from small unilamellar lipid vesicles in the fluid phase. To this end, we present two examples: (i) SAXS data for a (1:1) mixture of dioleoyl­phosphatidyl­choline and dioleoyl­phosphatidyl­ethanolamine, formed after extrusion through 30 nm membranes in ultra-pure water (Milli-Q), and (ii) a (1:1) mixture of dioleoyl­phosphatidyl­choline and dioleoylphosphatidylserine.

The simulation and FFT approach allows us to analyse the interplay between size polydispersity, vesicle shape fluctuations and curvature-dependent EDP. Since in the simulation we can control these different phenomena independently, we compare the relative effects of these phenomena on the distinct wavevector regions of the SAXS intensity, and thereby decouple their effect. The goal of our paper is to propose a strategy to analyse scattering data with respect to these three effects. Thereby, we address the limitations of current SUV SAXS analysis paradigms, namely idealization of one or more of the following effects: (i) polydispersity distribution of vesicle radii, (ii) thermal vesicle shape fluctuations and (iii) curvature dependence of the EDP. At the same time we can identify the range of parameters under which common ideal­izations are justified. In particular, we consider the validity of the factorization approximation of the SAXS intensity, *i.e.* the common assumption in analytical SAXS models that the scattering function can be described as a product of the bilayer form factor and the structure factor of the vesicle shape (Kiselev *et al.*, 2002 ▸, 2006 ▸). We hope that this contribution will facilitate the analysis of scattering data, which is particularly relevant given the currently increasing complexity in small-angle scattering experiments (Semeraro *et al.*, 2021 ▸). Beyond the specific effects considered here, we also want to develop the approach of simulating vesicle ensembles and subsequent 3D FFT in view of the analysis of more complex vesicles and vesicle shape transitions, as required for example in studies of synaptic vesicles. While here we primarily consider SAXS, the approach can equally be used for small-angle neutron scattering studies of vesicles, after minor modification regarding the scattering lengths.

## 2. Methods   

**Figure 1:**
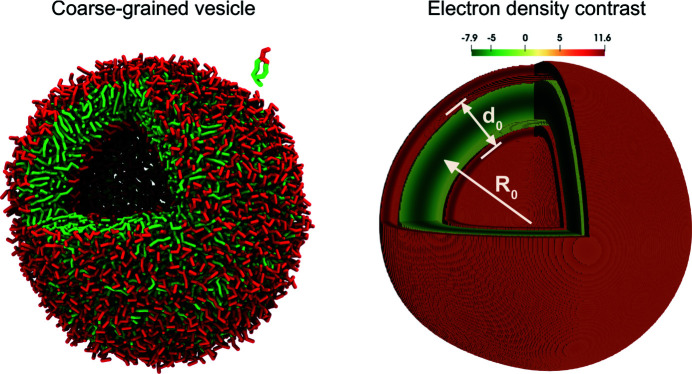
Hierarchical modelling of vesicles. (Left) The MARTINI coarse-grained model and (right) the continuum Helfrich model. Both vesicles have radius *R*_0_ = 8 nm, *i.e.* the distance from the centre of the vesicle to the middle of the membrane.

In the following we detail our hierarchical modelling approach, illustrated in Fig. 1 ▸. The system has two characteristic length scales – the larger one, *R*_0_ ≳ 10 nm, is associated with the vesicle radius and the smaller scale, *d*_0_ ≃ 4 nm, with the thickness of the membrane. We partition our model according to these two scales. The vesicle shape and its fluctuations are described by the Helfrich Hamiltonian (Helfrich, 1986 ▸; Milner & Safran, 1987 ▸) which characterizes a vesicle by its average size, *R*_0_, whose polydispersity obeys a distribution *P*(*R*_0_) and bending rigidity κ. The membrane structure and the electron density, ρ(*d*), in turn, characterize the small scale and are investigated by MD simulations of the coarse-grained MARTINI model (Marrink *et al.*, 2007 ▸). These simulations provide detailed density profiles across the bilayer, from which we extract the electron-density contrast that dictates the SAXS intensity. The two scales are coupled via the curvature dependence of the EDP.

First, we describe the study of the curvature-dependent membrane structure by MD simulations, comparing a planar membrane with a very small vesicle of radius ≲10 nm. Subsequently, we explain how we efficiently sample the large-scale shape fluctuations by simulating the Helfrich Hamiltonian, using an expansion of the radius in spherical harmonics of the polar and azimuthal angles. Finally, dressing the vesicle shape with a curvature-dependent EDP, we combine these two descriptions to calculate SAXS scattering curves.

### 2.1. MARTINI model simulation and EDPs   

#### 2.1.1. Simulation protocol   

**Figure 2:**
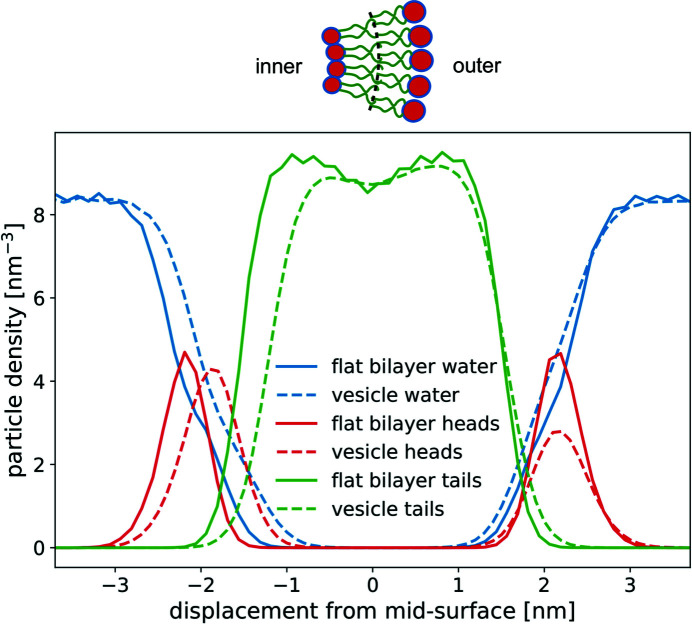
Plots of the number density of lipid heads (NC_3_ and PO_4_), tails (C1*A*–C4*A* and C1*B*–C5*B*) and water (*W*) versus displacement from the bilayer’s midplane for a planar membrane (solid lines) and highly curved vesicle (dotted lines). The schematic of a curved membrane at the top illustrates how lipid packing responds to curvature: lipid heads (smaller circles) in the inner leaflet are tightly packed, whereas lipid heads in the outer leaflet have a larger hydration shell (larger circles).

Coarse-grained MD simulations of a highly curved vesicle and planar lipid bilayer were performed using the *GROMACS* simulation package (Abraham *et al.*, 2015 ▸) in conjunction with the MARTINI force field (Marrink *et al.*, 2007 ▸). A small 1-palmitoyl-2-oleoyl-*sn*-glycero-3-phosphocholine (POPC) vesicle was formed by spontaneous aggregation, following the protocol of Risselada *et al.* (2008 ▸, 2014 ▸). The vesicle is composed of 1447 lipids in the outer leaflet and 770 lipids in the inner leaflet, embedded in a solvent that contains a total of 97 217 coarse-grained solvent particles. The vesicle radius is about 8 nm, which is twice as large as the membrane thickness, *d*_0_ = 4 nm. A planar bilayer patch was simulated at full hydration and contained 2048 POPC lipids. All systems were equilibrated in the *NPT* ensemble and simulated for 1 µs in the *NVT* ensemble at *T* = 300 K to calculate EDPs. The simulations provide detailed bead-type profiles across the planar or highly curved bilayer, as presented in Fig. 2 ▸.

#### 2.1.2. Electron-density profiles   

From these bead-type profiles we extracted the EDPs of the planar membrane and the highly curved vesicle. The numbers of electrons per coarse-grained bead are listed in Table 1 ▸ [see also Wanga *et al.* (2016 ▸)]. We used a simplified approach in which the centre of mass of the electron cloud coincides with the centre of mass of the coarse-grained bead.

**Figure 3:**
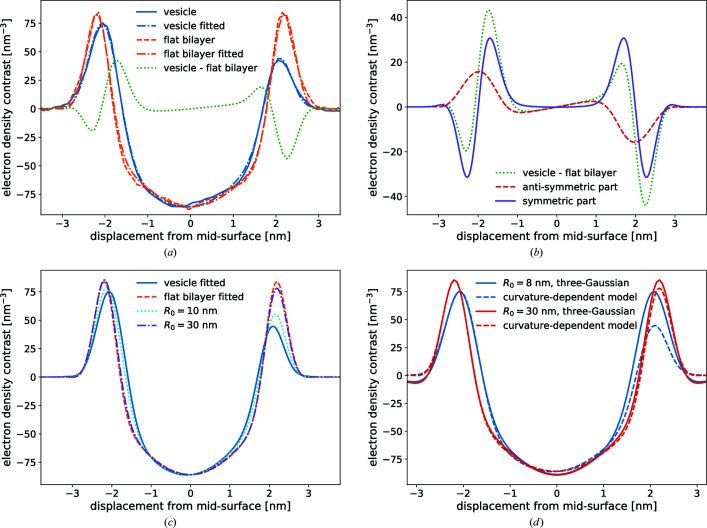
(*a*) Electron-density contrast of a planar bilayer patch (orange dashed line) and an 8 nm vesicle (blue solid line) of POPC lipids obtained by MD simulations, and fitted curves using equation (1) (dashed–dotted lines). The difference between vesicle and planar membrane electron densities is shown by the green dotted line. (*b*) Anti-symmetric, *d*_0_/*R*_0_ Δρ_a_(*d*) (red dashed line), and symmetric, (*d*_0_/*R*_0_)^2^Δρ_s_(*d*) (violet solid line), responses of the electron density to curvature (green dotted line). (*c*) Electron-density model of a vesicle of arbitrary radius *R*_0_ > 8 nm, as given by equation (2). (*d*) Electron-density contrast of vesicles with *R*_0_ = 8 nm and *R*_0_ = 30 nm obtained with the curvature-dependent model, equation (2) (blue and red dashed lines, respectively), and the curvature-independent three-Gaussian model, equation (4) (blue and red solid lines, respectively).

Fig. 3 ▸ summarizes the results from the coarse-grained MARTINI simulations. Fig. 3 ▸(*a*) presents the EDP of the POPC bilayer patch and the small vesicle of radius *R*_0_ = 8 nm, where we have subtracted the electron density of the water. We fitted these EDPs using two Gaussian functions and a fourth-order polynomial: 
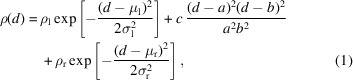
where *d* is the displacement from the bilayer’s midplane, and μ_l_ and μ_r_ denote the positions of the left (inner) and right (outer) peaks, respectively. These maxima in the electron density correspond to the head-group region of the bilayer membrane. σ_l_ and σ_r_ are the peak widths, and ρ_l_ and ρ_r_ characterize the peak heights. The polynomial fits the electron-density distribution in the bilayer’s hydrophobic interior. *a* and *b* are two roots of the fourth polynomial, and *c* is its value at the midplane, *d* = 0.

The parameters extracted by a nonlinear least-squares fit are compiled in Table 2 ▸. The fit was made with the Python package *SciPy* using the method optimize.curve_f it. The fit depends on the initial guess of the parameters and we selected parameters for the fourth polynomial such that the two real roots *a* and *b* are close to the vanishing tails of the electron density. The profiles of the planar membrane and highly curved vesicle differ mainly in two aspects: the heights and the positions of the two peaks. In the case of the planar membrane the EDP has two symmetric maxima, whereas for the highly curved vesicle these peaks are shifted closer to each other and differ in height. The higher peak corresponds to the inner monolayer of the small vesicle, where the lipid head groups are tightly packed because of the high curvature, whereas the outer monolayer is characterized by a lower head-group density due to the smaller curvature (Fig. 2 ▸). For the lipid tails the effect is opposite: when referred to the midplane of the bilayer, the area per molecule is lower in the inner monolayer of the vesicle than in the outer, and thus the volume density is also slightly lower in the inner tail region than in the hydrophobic portion of the outer monolayer. Additionally, the high curvature of the vesicle imparts a tension onto the membrane. This results in a thinning of the bilayer that is observable in the inwards shift of the position of the maxima of the electron density.

By virtue of symmetry, a model for the curvature dependence of the EDP of a vesicle can be written in the form of a curvature expansion: 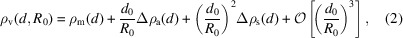
where *d* denotes the displacement from the centre of the vesicle’s midplane, and *d*_0_ and *R*_0_ are the bilayer thickness and vesicle radius, respectively. Note that this expansion of the EDP up to second order in curvature is compatible with the small-curvature expansion that underlies the Helfrich Hamiltonian, *i.e.* both the large-scale description of the vesicle shape and its fluctuations and the response of the small-scale bilayer structure to curvature include effects up to second order. ρ_m_ is the density profile of the planar bilayer, whereas the curvature dependence is described by Δρ_a_(*d*) and Δρ_s_(*d*) which are odd (anti-symmetric) and even (symmetric) functions with respect to their argument, *d*, respectively. The odd part describes the differences between the inner and outer monolayers in response to the membrane curvature, 1/*R*_0_. The even function, Δρ_s_(*d*), affects both monolayers in the same way, *e.g.* the curvature-induced thinning of the membrane. Since these effects do not depend on the sign of the curvature, they scale like 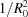 to leading order. If the symmetric contribution, Δρ_s_(*d*), chiefly stems from the curvature-induced thinning of the bilayer membrane, we can approximately relate its functional form to the profile of a planar bilayer by assuming that the profile of a bilayer of thickness *d*_0_ obeys the affine scaling relation 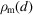 = 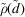 with the dimensionless 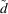 = 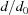. Thinning the bilayer by δ*d*_0_, we obtain for the change of profile
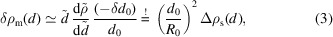

**Figure 4:**
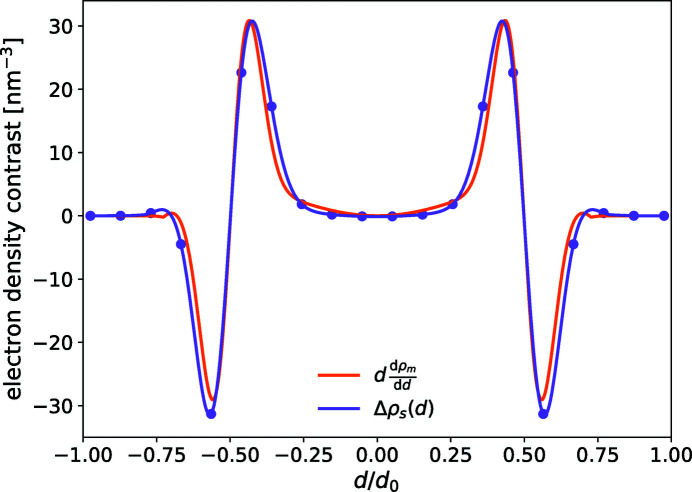
Electron-density contrast for the symmetric function, Δρ_s_, and the function *d*(dρ_m_/d*d*), plotted versus the rescaled parameter 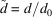, with *d*_0_ = 4 nm for the vesicle and 4.4 nm for the flat membrane. The discretization of the EDP used in the code is shown as points.

*i.e.* by symmetry, the thinning of the membrane scales like 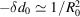 and the spatial dependence of the symmetric contribution is 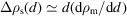. Fig. 4 ▸ includes a comparison between Δρ_s_(*d*), obtained by symmetrizing the difference ρ_v_(*d*, *R*_0_) − ρ_m_(*d*) for the vesicle with *R*_0_ = 8 nm, and 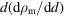, where the amplitude has been adjusted because δ*d*_0_(*R*_0_) is unknown. The good agreement indicates that (i) Δρ_s_ is, indeed, dominated by the curvature-induced thinning of the bilayer membrane and (ii) the affine scaling relation is appropriate for the range of thinning induced by the curvature.

Using this model we generated EDPs for vesicles with different radii and these are shown in Fig. 3 ▸(*c*). Note that for vesicle radii *R*_0_ ≲ 10 nm we observe significant deviations from the EDP of the planar bilayer.

A popular approximation for the EDP of vesicles is the sum of three Gaussian peaks (Brzustowicz & Brunger, 2005 ▸): 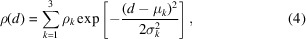
where the three maxima represent the head-group regions of the inner and outer monolayers, and the hydrophobic membrane interior, respectively. As in equation (1), the parameters ρ_*k*_, μ_*k*_ and σ_*k*_ characterize the height, position and widths of the *k*th peak, respectively. The electron density of this three-Gaussian model with symmetric inner and outer maxima is compared in Fig. 3 ▸(*d*) with the curvature-dependent model [equation (2)] for a small vesicle, *R*_0_ = 30 nm, and a highly curved vesicle, *R*_0_ = 8 nm. The parameters of the symmetric model [equation (4)] have been adjusted to fit the maximum of the electron density of the inner monolayer and the parameters of the fit are listed in Table 3 ▸. This comparison illustrates that our curvature-dependent model and the symmetric three-Gaussian model provide a similar representation of the electron density for large radii, *R*_0_ ≳ 30 nm. Adjusting the parameters of the symmetric three-Gaussian model to our curvature-dependent model, the electron-density maxima coincide (by construction), but the electron-density contrast is slightly underestimated in the tail region and at the interface between the head groups and the solvent. For smaller radii, *R*_0_ ≲ 10 nm, however, the two models for the electron density differ significantly because the asymmetry of the profile becomes important.

### 2.2. Generation of vesicle shapes and SAXS scattering intensity   

#### 2.2.1. Size polydispersity and shape fluctuations   

In order to obtain the electron density of a vesicle in 3D space, we convolute the position of the bilayer’s midplane with the curvature-dependent electron-density contrast discussed in the previous section. Apart from translations, the position of the bilayer’s midplane is dictated by the size of the vesicle and the thermal fluctuations of its shape. In order to consider size polydespersity we assume the vesicle sizes *R*_0_ are Gaussian distributed, according to
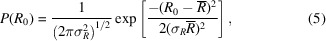
where 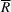 characterizes the mean vesicle radius of the ensemble and 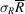 denotes the (dimensional) standard deviation. The latter quantity characterizes the radius polydispersity. The Gaussian distribution is a common approximation for an equilibrated size distribution. Our computational scheme, however, can be straightforwardly generalized to different distributions.

Given the vesicle radius *R*_0_, thermal fluctuations will result in deviations from a spherical shape. The free-energy costs of deviations from a spherical shape are proportional to the bending rigidity, κ, and quantified by the Helfrich Hamiltonian. In the following we assume that the thermally excited deviations from the spherical shape remain small and we parameterize the position of the bilayer’s midplane by the distance *R*(θ, ϕ) from the vesicle’s centre of mass. In this spherical coordinate system, we expand *R*(θ, ϕ) in terms of spherical harmonics, 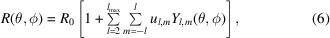
with 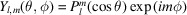, where 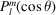 are associated Legendre polynomials. Thus the shape of a vesicle, *i.e.* the position of the bilayer’s midplane up to translation, is characterized by *R*_0_ and the set of *u*_*l*,*m*_. In thermal equilibrium, the Helfrich Hamiltonian asserts that the fluctuation amplitudes, *u*_*l*,*m*_, are statistically independent and Gaussian distributed with zero mean and variance 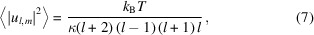
where κ is the bending rigidity of the membrane. The case where *l* = 1 merely corresponds to a translation of the centre of mass and is therefore omitted. We consider modes up to *l*_max_ = 6. In order to resolve shape fluctuations on the vesicle with a wavelength that is comparable to the membrane thickness *d*_0_ – the smallest wavelength where the Helfrich Hamiltonian is applicable – the order of spherical harmonics *l*_max_ should be chosen such that the ratio 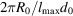 is of order unity.

The assumption of small deviations from a spherical shape in conjunction with the parameterization *via* spherical harmonics allows us to generate independent equilibrated vesicle shapes by (i) choosing a radius *R*_0_ according to equation (5) and (ii) drawing the coefficients *u*_*l*,*m*_ from Gaussian distributions with widths given by equation (7). The configurations thus generated are uncorrelated, *i.e.* unlike molecular simulations of particle-based models or simulations of dynamically triangulated surfaces we obtain a new membrane shape at each generation step.

#### 2.2.2. Calculation of SAXS scattering intensity   

In order to compute the SAXS scattering intensity from an ensemble of vesicles with different sizes and shapes, we combine the generation of configuration snapshots of the location of the membrane’s midplane with the curvature-dependent EDPs. The electron density in 3D space is obtained by
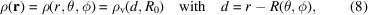
where ρ_v_(*d*, *R*_0_) is the curvature-dependent EDP according to equation (2) and *R*(θ, ϕ) denotes the local distance of the bilayer’s midplane from the vesicle’s centre of mass [equation (6)]. This procedure neglects (i) the difference between the local curvature of the bilayer’s midplane and the inverse size, 1/*R*_0_, of the vesicle, and (ii) the difference between the local normal of the bilayer’s midplane and the radial vector to the vesicle’s centre of mass. Both approximations are consistent with the assumed small deviation of the fluctuating vesicle from its lowest energy state, a spherical vesicle.

In order to calculate the scattering intensity, we collocalize the electron density, ρ(**r**), on a regular cubic grid with *N* × *N* × *N* sites. Typically, we use *N* = 400 or 600 in each Cartesian direction, and the spatial extent of the collocation grid is *L* = 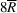. Thus the collocation grid can resolve spatial distances 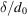 = 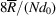. For small vesicles, with 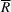 = 8, 10 and 20 nm, we use *N* = 400, resulting in δ/*d*_0_ = 0.1 for 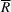 = 20 nm. The same resolution is obtained for the largest vesicle, 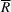 = 30 nm, for *N* = 600. There are two characteristic length scales, 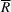 and *d*_0_, and we choose the dimensionless wavevector 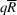 to present our results. The scattering intensity is numerically obtained by FFT of the electron density ρ(**r**) on the regular cubic grid according to 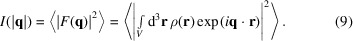
The average 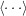 runs over all orientations of scattering vectors **q** – the ‘powder average’ – as well as independent snapshots of vesicle shapes. We use *N*_v_ = 1000 independent snapshots of the vesicle shape to compute the scattering intensity. The simulations were run on a parallel cluster with Ivy-Bridge Intel E5-2670 v2 CPUs, 2.5 GHz 2 × 10 cores and 64 GB memory, requiring 2.5 s per vesicle per core. To compare the deviation between two scattering intensities, *I*(*q*) and *I*_0_(*q*), we use the mean-squared variance: 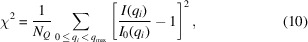
where *N*_*Q*_ denotes the number of *q* values in the considered interval.

## 3. Results and discussion   

### 3.1. Structure factor and form factor   

To understand qualitatively the features of the scattering intensity *I*(*q*), we factorize *I*(*q*) into a structure factor, which describes the shape of the vesicle and its fluctuations, and a form factor of the membrane, which contains information about the EDP of the bilayer membrane (Kiselev *et al.*, 2002 ▸). Such a factorization approximation is justified if the two length scales, vesicle radius *R*_0_ and bilayer thickness *d*_0_, are well separated, *i.e.* 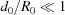. Using the assignment of equation (8), we obtain for the scattering amplitude of a vesicle with shape *R*(θ, ϕ)
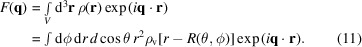
The scattering intensity is obtained by averaging over the orientations of *q* and the vesicle shapes [equation (9)]. For completeness, we recall the steps that result in the factorization approximation (Kiselev *et al.*, 2002 ▸).

(i) In the absence of size polydispersity and thermal fluctuations, the vesicle shape is simply a sphere of radius *R*(θ, ϕ) = *R*_0_, and we obtain
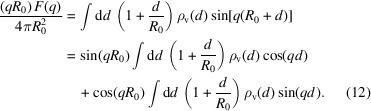
(ii) According to equation (2), the EDP ρ_v_(*d*) is a sum of even and odd contributions. In the following, we keep all terms up to second order in the vesicle’s curvature, *d*_0_/*R*_0_: 
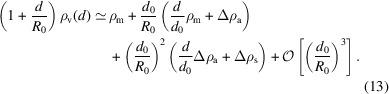



Inserting this expansion into equation (12), we obtain for the scattering amplitude of a thin spherical vesicle
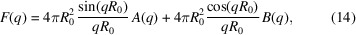


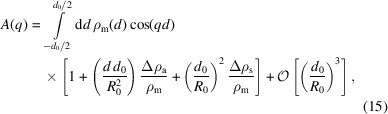


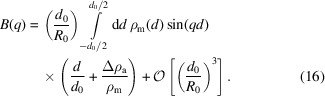



Using this expression we obtain for the scattering intensity *I*(*q*) up to first order in curvature
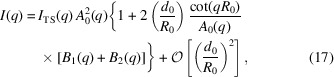


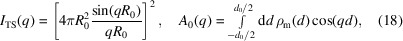


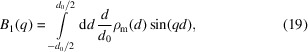


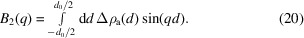
The leading term is the popular factorization approximation, where *I*_TS_ is the structure factor of an infinitely thin spherical shell and 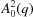 is the powder average of the planar bilayer form factor. Equation (18) indicates under which conditions the factorization approximation is accurate.

**Figure 5:**
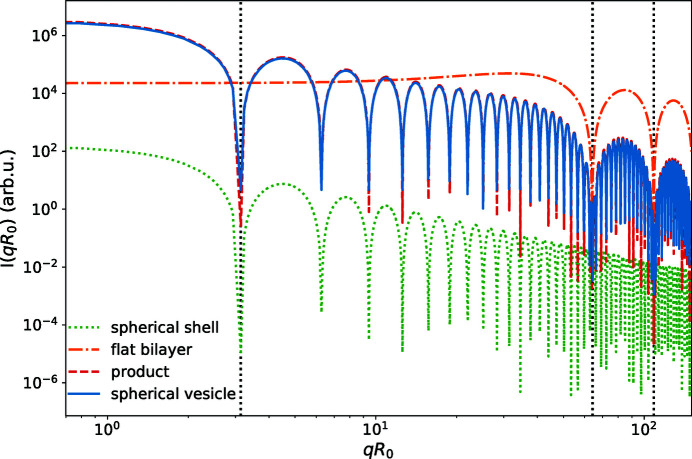
Scattering intensity of an infinitely thin spherical shell of *R*_0_ = 30 nm (green dotted line), a planar membrane bilayer, *R*_0_ = ∞, of thickness *d*_0_ = 4 nm (orange dashed–dotted line), the product of both (red dashed line) and simulation results for a spherical vesicle of finite width, 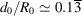, with the curvature-dependent EDP (solid blue line).

The structure factor of a spherical shell and the powder-averaged form factor of a planar bilayer are depicted in Fig. 5 ▸ for *R*_0_ = 30 nm and *d*_0_ = 4 nm. Even for this vesicle size, one can clearly observe the separation of the two length scales *R*_0_ and *d*_0_. Most notably, the radius of the vesicle can simply be obtained from the wavevector, *q*_v_ = π/*R*_0_, at which the structure factor vanishes. The form factor of the planar bilayer in turn, 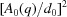, is only a function of *qd*_0_ and basically remains wavevector independent for *q* ≲ *q*_m_ = 2π/*d*_0_. Fig. 5 ▸ also depicts the result for a spherical vesicle with curvature-dependent EDP. The curvature dependencies, Δρ_a_ and Δρ_s_, and the first-order correction term 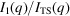 = 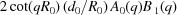 appear to be negligibly small, even for 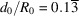.

### 3.2. Models of electron-density profiles   

In this part we will analyse the effect of different EDPs on the scattering intensity. The scattering amplitude of an ideally spherical vesicle with radius *R*_0_, whose EDP is given by three Gaussian peaks [equation (4)], takes the form (Brzustowicz & Brunger, 2005 ▸)
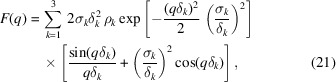
where δ_*k*_ = *R*_0_ + μ_*k*_ denotes the distance of the head-group and tail regions from the centre of mass of the vesicle. We consider an asymmetric EDP, where the head-group peaks differ in their amplitudes, ρ_1_ ≠ ρ_3_. They are assumed (i) to be symmetrically displaced by a distance μ = −μ_1_ = μ_3_ from the bilayer’s midplane, μ_2_ = 0, and (ii) to have the same width, σ = σ_1_ = σ_3_. Moreover, we assume that (iii) the widths of the Gaussian peaks and the bilayer thickness are small compared with the vesicle’s radius, 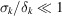 and 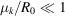, respectively. Because of condition (iii) we expand equation (21) up to second order in σ_*k*_/*R*_0_ and μ_*k*_/*R*_0_ and obtain
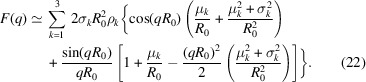



**Figure 6:**
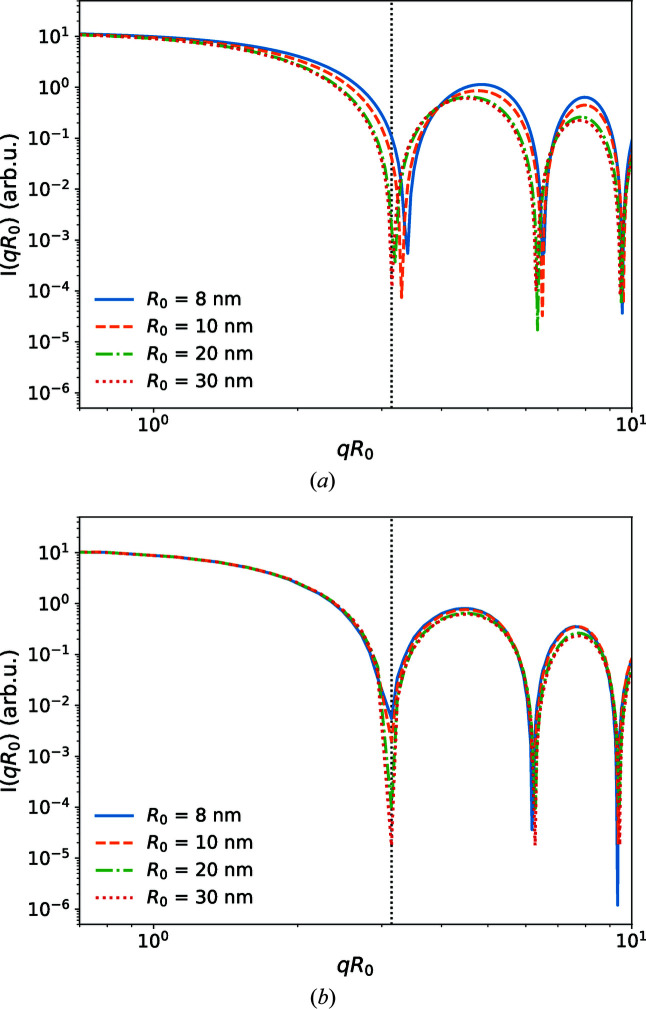
Scattering intensity *I* as a function of the dimensionless wavevector *qR*_0_ obtained (*a*) from the symmetric three-Gaussian EDP with ρ_1_ = ρ_3_ [*cf*. equation (4) and Table 3 ▸] and (*b*) with the curvature-dependent EDP, equation (2), parameterized from the MARTINI model simulation (*cf*. Fig. 3 ▸).

For giant vesicles (or, equivalently, extremely thin bilayer membranes), μ_*k*_/*R*_0_ → 0 and σ_*k*_/*R* → 0 and equation (22) predicts 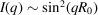, *i.e.* the first minimum of the scattering intensity occurs at *qR*_0_ = π, in agreement with the factorization approximation. Fig. 6 ▸(*a*) shows the scattering intensity for vesicles of different radii *R*_0_ and a bilayer thickness of *d*_0_ = 4 nm for an EDP given by the symmetric three-Gaussian model, *i.e.* ρ_1_ = ρ_3_. The parameters are compiled in Table 3 ▸. We indeed observe that the first minimum is close to *q*_v_ = π/*R*_0_ for *R*_0_ = 30 nm. For smaller values of *R*_0_ comparable to the bilayer thickness, *d*_0_ ≃ 2μ, this minimum is shifted to larger wavevectors, *i.e.* estimating the vesicle radius from the first minimum of the scattering intensity will underestimate *R*_0_. This shift of the minimum position, *q*_v_ *R*_0_ − π, scales as μ/*R*_0_. Fig. 6 ▸(*b*) presents plots for ideally spherical vesicles with radius *R*_0_ using our curvature-dependent EDP [equation (2)] that has been parameterized from the MARTINI model simulations. The main difference from the symmetric three-Gaussian model is the asymmetry of the inner and the outer electron-density maxima of the head groups, as displayed in Fig. 3 ▸(*d*). Most notably, there is no discernible shift in the location of the first minimum as a function of *qR*_0_, *i.e.* the first minimum remains an accurate estimate of the vesicle’s radius even for small vesicles. Apparently, corrections to the factorization approximation and curvature-induced asymmetry of the EDP cancel out. Using equation (22) which describes the scattering intensity of the asymmetric three-Gaussian model up to second order in curvature, we can determine which asymmetry, ρ_1_ ≠ ρ_3_, results in such a cancellation. Such a cancellation occurs if the coefficient in front of the cosine term of equation (22) vanishes, *i.e.*

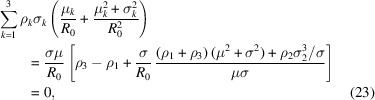


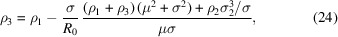

*i.e.* the necessary asymmetry of the EDP is proportional to the curvature of the vesicle. Clearly, the electron density ρ_3_ of the head groups in the outer monolayer located at *r* = *R*_0_ + μ is smaller than ρ_1_ of the inner monolayer at *r* = *R*_0_ − μ, in agreement with the curvature-dependent electron-density model parameterized by results of the MARTINI model simulations.

### 3.3. Distinction of thermal fluctuations and size polydispersity   

**Figure 7:**
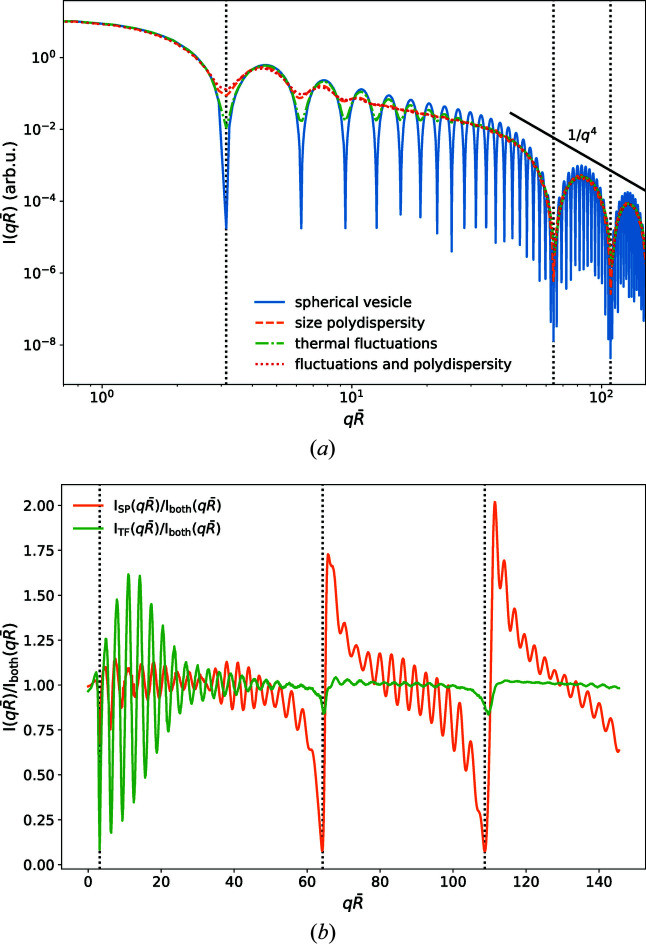
(*a*) Simulated SAXS scattering intensity *I*(*q*) of an ideally thin spherical vesicle of radius *R*_0_ = 30 nm (solid blue line), an ensemble of polydisperse vesicles σ_*R*_ = 0.1 (orange dashed line), an ensemble of thermally fluctuating vesicle shapes κ = 10*k*_B_ *T* and *l*_max_ = 6 (green dashed–dotted line), and an ensemble with both size polydispersity and thermal fluctuations (red dotted line). (*b*) Ratio of the simulated scattering intensity of polydisperse but purely spherical vesicles *I*_SP_(*q*) (orange, σ_*R*_ = 0.1) and of vesicles showing only thermal fluctuations *I*_TF_(*q*) (green, κ = 10*k*_B_ *T*) to the intensity of an ensemble with both contributions, *I*_both_(*q*).

Fig. 7 ▸(*a*) presents the scattering intensities obtained for ideally spherical vesicles with fixed radius, *R*_0_ = 30 nm, ideally spherical vesicles with polydisperse radii using the distribution [equation (5)] with σ_*R*_ = 0.1, vesicles with thermal shape fluctuations characterized by κ = 10*k*_B_ *T* and *l*_max_ = 6, and vesicles that have both a polydisperse radius *R*_0_ and thermal shape fluctuations. We observe that both size polydispersity and shape fluctuations smooth out the features of *I*(*q*), in particular the first minimum at *q*_v_. In experiments, the ideal­ization of an ensemble of monodisperse ideally spherical vesicles breaks down – due to the limitations of experimental vesicle preparation, the vesicle sizes are polydisperse, and the thermal fluctuations that give rise to shape fluctuations are a hallmark of soft matter (Székely *et al.*, 2010 ▸; Seifert, 1997 ▸). Fig. 7 ▸(*b*) presents the relative effects of size polydispersity and vesicle shape fluctuations on the simulated SAXS curves. To highlight the differences, we show the ratio 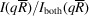 of the scattering intensity with either only thermal fluctuations or only size polydispersity to a reference curve including both phenomena. This representation corresponds to the case where, if it were possible in an experiment, one or other of these phenomena could be ‘switched off’. In the absence of polydispersity, *i.e.* for thermally fluctuating vesicle shapes with constant *R*_0_, the ratio exhibits rapidly decaying oscillations that are only sizable in the wavevector range 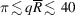, *i.e.* switching off polydispersity in the simulation chiefly affects the small-*q* regime, in accordance with the expectation that vesicle size is the largest characteristic length scale. In the absence of thermal shape fluctuations, *i.e.* for ideally spherical vesicles of varying size, we observe that the rapid oscillations are modulated by a sawtooth pattern that extends to much larger *q* vectors, comparable to the minima of the bilayer’s form factor, 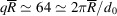. Again, this corroborates the expectation that thermal fluctuations also affect the smaller length scales.

**Figure 8:**
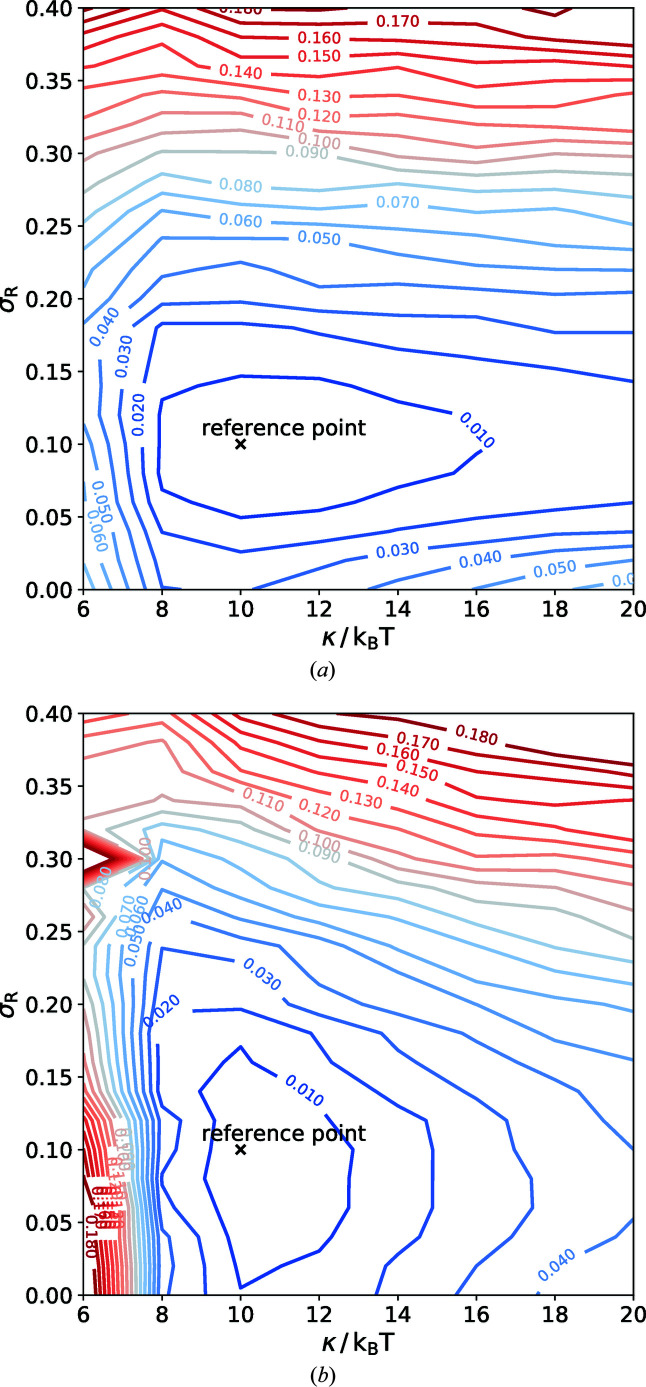
Isocontour plots of χ^2^ of simulated intensities for different poly­dispersities σ_*R*_ and bending rigidities κ/(*k*_B_ *T*), calculated using equation (10) with (*a*) 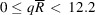 and (*b*) 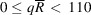.

As illustrated above, size polydispersity and thermal shape fluctuations give rise to small-*q* and large-*q* signatures in the scattering intensity. In order to gauge the ability to extract the dimensionless parameters σ_*R*_ and κ/*k*_B_ *T* that characterize the size distribution and the strength of thermal fluctuations, respectively, from the scattering intensity, we generated scattering data with known values of 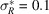, 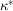/*k*_B_ *T* = 10 and 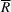 = 30 nm, and compared the result with scattering intensities at other parameter doublets (σ_*R*_, κ/*k*_B_ *T*). This illustrates how sensitively the scattering intensity depends on the two parameters and identifies correlations in their estimates. As before, the scattering intensity is computed from *N*_v_ = 1000 independent vesicle configurations. Fig. 8 ▸ shows a contour plot of the χ^2^ deviation [see equation (10)]. In panel (*a*) the comparison of the scattering intensities is extended over the low-*q* wavevector regime 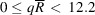, whereas panel (*b*) displays the comparison for an extended interval of wavevectors, 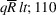. In both cases, there appear to be no significant correlations between the estimates of σ_*R*_ and κ/*k*_B_ *T*, *i.e.* in the vicinity of the true values 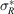 and 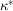/*k*_B_ *T*, the minor and major axes of the elliptical contour lines of constant χ^2^ are aligned with the σ_*R*_ and κ/*k*_B_ *T* axes in Fig. 8 ▸. Extending the wavevector regime to large *q* significantly increases the accuracy of the estimate of the bending rigidity but does not significantly affect the estimate for the relative variance σ_*R*_ of the vesicle size distribution. This observation corroborates the discussion of Fig. 7 ▸.

### 3.4. Application to experimental data   

Now we use the approach presented above for least-squares fitting of experimental vesicle SAXS data. First, we demonstrate that simulating an ensemble of vesicles and computing the structure factor by 3D FFT followed by radial averaging can be implemented practically. Second, we test whether the effects of thermal fluctuations and/or static vesicle shape deformations are relevant, in the sense that the achievable experimental data quality suffices to distinguish these effects. Third, we compare the fitting parameters for the bilayer EDP obtained from the new approach with a conventional analytical model.

To this end, we compare our method with the vesicle SAXS model of Brzustowicz & Brunger (2005 ▸), which is commonly used for SAXS analysis. This model uses a sum of *N* Gaussians with amplitude ρ_*i*_ and width σ_*i*_ to describe ρ_m_. It assumes spherical symmetry for the vesicle shape and, in addition to the treatment of Kiselev *et al.* (2002 ▸) described in Section 3.1, also accounts for polydispersity of the vesicle sizes by a Gaussian distribution with standard deviation σ_*R*_. The latter is integrated analytically, with the integration limits being set to ± ∞ – an approximation which breaks down at high relative polydispersity σ_*R*_ ≃ *R*_0_. Furthermore, the approximation 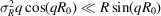 is employed to obtain the final analytical function for least-squares fitting, 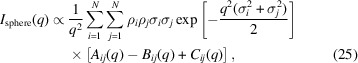
where the wavevector-dependent coefficients *A*_*ij*_(*q*), *B*_*ij*_(*q*) and *C*_*ij*_(*q*) are given by 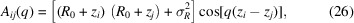


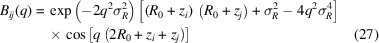
and 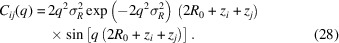



For least-squares fitting of experimental data, the present approach based on simulating fluctuating or deformed vesicles and subsequent Fourier transformation (3D FFT), is used as follows. As in the work of Brzustowicz & Brunger (2005 ▸), we use the separated-form-factor (SFF) approximation and model the EDP ρ_m_ in terms of three Gaussians. For all fits we also impose mirror symmetry of the EDP, neglecting curvature-induced effects, since *R*_0_ ≫ *d*_0_. Making use of the SFF approximation, we can hence factor out *F*(*q*) in the fitting function and simulate vesicles with a box profile of constant width 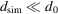. The simulated ‘δ-vesicle’ hence captures only the vesicle shape, not its EDP, significantly reducing the simulation runs. For each κ, we simulate *N* = 1000 vesicles for constant (unit) radius to compute and tabulate the vesicle structure factor *S*(*Q*) in natural units *Q* = *qR*_0_. These tabulated data are scaled in the fitting function with the fitting parameter *R*_0_, using interpolation for the sampling points in both *q* and κ. In the same manner, if applicable, we model a static vesicle shape deformation, 〈*u*_*l*, *m*_〉 ≠ 0 for *l* = 2, *m* = 0, to represent a nonspherical, oblate or prolate average vesicle shape.

Two different lipid vesicle data sets are used for these tests: (*a*) a (1:1) mixture of dioleoylphosphatidylcholine (DOPC) and dioleoylphosphatidylethanolamine (DOPE), formed after extrusion through 30 nm membranes in ultra-pure water (Milli-Q), and (*b*) a (1:1) mixture of DOPC and dioleoylphosphatidylserine (DOPS), which was also formed in ultra-pure water but then immersed in 4 m*M* glucose solution to induce osmotic pressure and vesicle deformation. Note that it is well known from phase diagrams of vesicle shapes that a surplus of vesicle surface over volume results in a transition from a spherical to a prolate shape (Seifert *et al.*, 1991 ▸; Seifert, 1997 ▸). SAXS data were collected on the bending magnet beamline BM29 (BioSAXS) at the European Synchrotron Radiation Facility (ESRF) in Grenoble, France, at photon energy *E* = 12.5 keV using a multilayer monochromator with Δ*E*/*E* ≃ 0.01, and a pixel detector (Pilatus 1M, Dectris) at a sample-to-detector distance of 2.867 m to cover a *q* range of approximately 0.036–4.95 nm^−1^. The sample suspension was automatically loaded into a vacuum-mounted quartz capillary of 1.8 mm in diameter for exposure to the beam. For details of the sample preparation, experiment and data correction we refer readers to the article by Komorowski *et al.* (2018 ▸), where the first of the two curves was published (with a standard fitting workflow).

**Figure 9:**
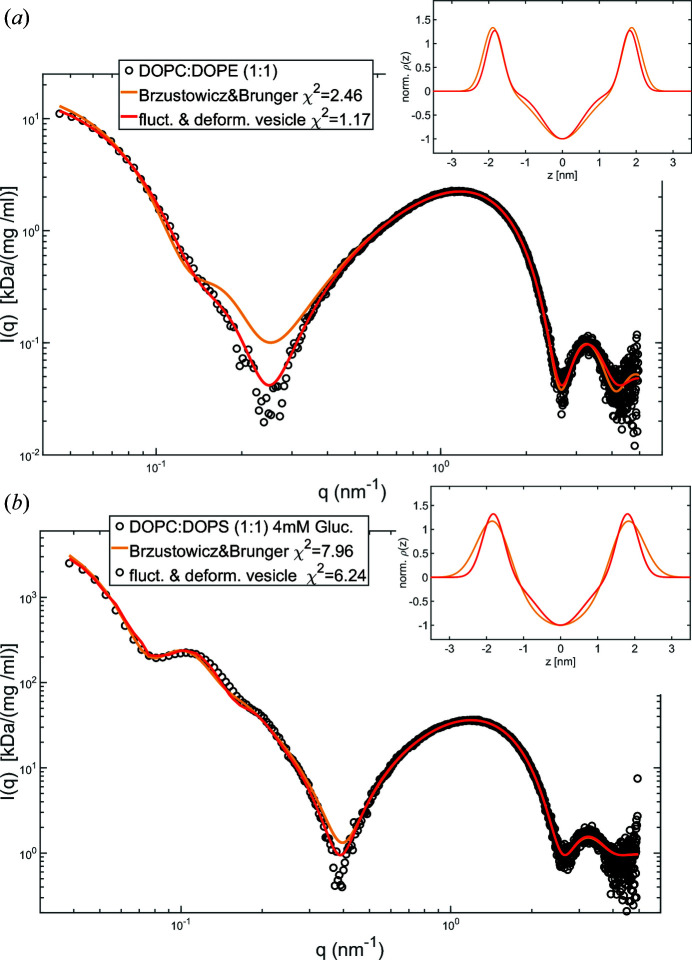
(*a*) SAXS data for 30 nm extruded DOPC:DOPE (1:1) vesicles in ultra-pure water (black circles), with least-squares fits (solid lines) to the analytical model according to Brzustowicz & Brunger (2005 ▸) (orange) and to simulated and tabulated scattering curves computed for fluctuating vesicles according to the present method (red). The inset shows the respective EDPs, in both cases modelled by three Gaussians (head group, tail region, head group). (*b*) SAXS data for DOPC:DOPS (1:1) vesicles suspended in 4 m*M* glucose solution (black circles) to induce a vesicle shape deformation by osmotic pressure. Again, the fit to the Brzustowicz & Brunger model (orange) is compared with the present approach (red), and the corresponding EDPs are shown in the inset. The fitting parameters are summarized in Table 4 ▸.

Fig. 9 ▸ shows the least-squares fitting of experimental SAXS curves, by the present approach (red) as well as by the Brzustowicz & Brunger model (orange), for the extruded vesicles of (*a*) the equimolar DOPC:DOPE mixture and (*b*) the equimolar DOPC:DOPS mixture in 4 m*M* glucose solution. In both cases the simulation/FFT fit captures in particular the region around the first form-factor minimum better, resulting in a smaller norm of the residual (reduced χ^2^). This effect is particularly pronounced for the DOPC:DOPE data. Here κ = 7*k*_B_ *T* provides the best fit.

For the DOPC:DOPS system, the static deformation *u*_2,0_ was varied to account for the expected shape transition due to osmotic pressure. Indeed, the results indicate a transition to a prolate shape (*u*_2,0_ > 0) after the vesicle volume decreases as a result of water permeation induced by the osmotic gradient, in line with the theoretical phase diagram (Seifert *et al.*, 1991 ▸; Seifert, 1997 ▸). Note also that the EDPs obtained from the fits using the current model are more plausible (red versus orange profiles in the inset), *i.e.* they exhibit in particular a higher head-group density and a smaller width, more similar in shape to those obtained from multilamellar planar mem­branes. All fitting parameters are presented in Table 4 ▸.

Thus, while we clearly see that polydispersity masks vesicle shape effects to a large extent, a residual benefit of our model is found in terms of χ^2^ (at the cost of one extra fitting parameter), and some residual sensitivity for the effects of interest remains. For preparation methods yielding smaller σ_*R*_, such as purification or size fractionation with high-performance liquid chromatography columns, and for softer bilayers, the effects would be correspondingly stronger.

## 4. Conclusions   

In this paper we have analysed, by means of computer simulations, the curvature dependence of the electron-density profile of small unilamellar vesicles, as well as the effects of thermal fluctuations and size polydispersity on small-angle scattering curves. Curvature changes the equilibrium bilayer structure, as captured by coarse-grained mol­ecular dynamics simulations, and results in a thinning of the inner leaflet and a decrease in the head-group density of the outer leaflet. Hence the EDP becomes asymmetric, even for a membrane with the same lipid composition in the inner and outer leaflets. This curvature effect becomes relevant for radii *R*_0_ < 30 nm, which can be encountered in experimental preparations of extruded or sonicated lipid vesicles, as well as in biological compartments such as synaptic vesicles. Importantly, the curvature-induced asymmetry is high enough that it may be observed experimentally in future for very small vesicles. Highly resolved experimental bilayer profiles could thus, in principle, also provide information on the interplay of asymmetric lipid partitioning and curvature. To this end, we have also investigated how the small-angle scattering distribution changes for small *R*_0_ ≲ 10 nm when the common factorization approximation breaks down. In this regime, the scattering function can no longer be modelled as the product of a powder-averaged bilayer form factor and the transform of the vesicle shape, for example a thin hollow sphere. We find that for a symmetric bilayer the first minimum of the scattering function is positively shifted with respect to the value *q*_v_ = π/*R*_0_, which underestimates the vesicle radius for small vesicles. Interestingly, this shift is eliminated for curvature-induced bilayer asymmetry. Hence, the curvature-dependent electron-density model fixes the position of the first minimum of the scattering intensity at *q*_v_ = π/*R*_0_, despite the fact that the factorization approximation is invalid. In summary, the curvature corrections become important for very small vesicles, resulting, for example, in a 20% density difference between inner and outer leaflets for *R*_0_ ≲ 10 nm. In contrast, for vesicles with *R*_0_ ≳ 30 nm the curvature has very little effect and, at the same time, the factorization approximation becomes valid.

In addition to curvature, we have studied the effects of size polydispersity and thermal fluctuations, which both result in a smearing out of the scattering curve minima. However, the exact functional forms differ. In fact, polydispersity and thermal fluctuations modify the scattering curve in different *q* regimes: thermal fluctuations predominantly affect the scattering intensity around the membrane form-factor minima, corresponding to the membrane structure, while the vesicle size polydispersity mainly contributes in the small-*q* range, reflecting the vesicle size. Hence, our results show that thermal fluctuations are not completely masked by polydispersity, which is almost always unavoidable in experiments. At the same time, this study also explains when the classical vesicle models that assume perfect spherical symmetry work relatively well. However, if further progress is made in purification or size fractionation, more details of the shape fluctuations will become visible and should be accounted for. This also holds for small changes in the average vesicle shape, which are sensitive indicators for changes in vesicle volume and membrane area.

More generally, we have presented an approach which helps to free experimental investigations of vesicles from idealizing assumptions, by directly modelling and simulating vesicle structures for the relevant parameters, and calculating the scattering function based on 3D FFT on a suitable numerical grid. Since this approach is computationally efficient, it is also suitable for the analysis of experimental SAXS data. In future, it could easily be augmented to accommodate either more complicated configurations, including adhering vesicles, or vesicles with lipids and large membrane proteins. In particular, it could be used to study the structure of synaptic vesicles, including the effects of protein clusters and/or shape transitions. All of the above-mentioned systems can be modelled on a numerical grid, while the 3D FFT and radial averaging provide the link to the experimental data.

## Figures and Tables

**Table 1:** Number of electrons per coarse-grained bead of the POPC lipid

Bead	NC_3_	PO_4_	GL1	GL2	C1*A*	C2*A*	C3*A*
No. of electrons	49	56	29	30	30	30	30
							
Bead	C4*A*	C1*B*	C2*B*	D3*B*	C4*B*	C5*B*	*W*
No. of electrons	31	27	27	27	27	27	40

**Table 2:** Fitting parameters of equation (1) for the EDP for a planar bilayer and a highly curved vesicle (*R*_0_ = 8 nm)

	ρ_l_ (nm^−3^)	μ_l_ (nm)	σ_l_ (nm)	*a* (nm)	*b* (nm)	*c* (nm^−3^)	ρ_r_ (nm^−3^)	μ_r_ (nm)	σ_r_ (nm)
Bilayer	108.4	−2.2	0.27	−3.2	3.2	−85.9	108.4	2.2	0.27
Vesicle	107.4	−2.0	0.35	−3.2	3.0	−86.0	168.1	2.0	0.32

**Table 3:** Parameters of the symmetric three-Gaussian model [equation (4)], shown in Fig. 3 ▸(*d*) ρ_1_ = ρ_3_, μ = −μ_1_ = μ_3_, σ_1_ = σ_3_ and μ_2_ = 0.

*R*_0_ (nm)	ρ_1_ (nm^−3^)	μ (nm)	σ_1_ (nm)	ρ_2_ (nm^−3^)	σ_2_ (nm)
10	107.0	2.10	0.33	−88.0	1.40
20	109.0	2.16	0.29	−89.1	1.34
30	111.3	2.17	0.28	−89.2	1.38

**Table 4:** Fitting parameters obtained by least-squares fitting of the SAXS data for 30 nm extruded DOPC:DOPE (1:1) vesicles and for 100 nm extruded DOPC:DOPS (1:1) vesicles + 4 m*M* glucose (Fig. 9 ▸) The structural bilayer parameters are ρ_h_ = ρ_h1_ = ρ_h2_ and σ_h_ = σ_h1_ = σ_h2_ for a symmetric bilayer profile. The amplitude of the Gaussian representing the chain region is set to ρ_c_ = −1, *i.e.* the electron-density difference from water is fitted (shown in Fig. 9 ▸, insets) and normalized to the electron-density difference between water and the bilayer centre plane. *u*_2,0_ is the static deformation.

Sample	Model	ρ_h_ (a.u.)	σ_h_ (nm)	σ_c_ (nm)	*d*_hh_ (nm)	*R*_0_ (nm)	σ_*R*_ (nm)	κ	*u*_2,0_	\chi_{\rm red}^2
DOPC:DOPE (1:1)	Fluctuating vesicle	1.28	0.23	0.53	3.67	23.54	4.83	7*k*_B_ *T*		1.17
	Spherical vesicle	1.35	0.26	0.63	3.77	17.62	6			2.46

DOPC:DOPS (1:1) + 4 m*M* glucose	Fluctuating vesicle	1.34	0.29	0.63	3.62	47.1	6.2		0.9	6.24
	Spherical vesicle	1.33	0.44	0.94	3.6	36.7	8			7.96
